# Investigations for Thermal and Electrical Conductivity of ABS-Graphene Blended Prototypes

**DOI:** 10.3390/ma10080881

**Published:** 2017-07-31

**Authors:** Rupinder Singh, Gurleen S. Sandhu, Rosa Penna, Ilenia Farina

**Affiliations:** 1 Department of Production Engineering, Guru Nanak Dev Engineering College, Ludhiana 141006, India; gurleensandhu91@gmail.com; 2 Department of Engineering, University of Salerno, 84084 Fisciano, Italy; rpenna@unisa.it; 3 Department of Engineering, University of Naples Parthenope, 80143 Naples, Italy; ilenia.farina@uniparthenope.it

**Keywords:** thermal conductivity, electrical conductivity, FDM, graphene, ABS

## Abstract

The thermoplastic materials such as acrylonitrile-butadiene-styrene (ABS) and Nylon have large applications in three-dimensional printing of functional/non-functional prototypes. Usually these polymer-based prototypes are lacking in thermal and electrical conductivity. Graphene (Gr) has attracted impressive enthusiasm in the recent past due to its natural mechanical, thermal, and electrical properties. This paper presents the step by step procedure (as a case study) for development of an in-house ABS-Gr blended composite feedstock filament for fused deposition modelling (FDM) applications. The feedstock filament has been prepared by two different methods (mechanical and chemical mixing). For mechanical mixing, a twin screw extrusion (TSE) process has been used, and for chemical mixing, the composite of Gr in an ABS matrix has been set by chemical dissolution, followed by mechanical blending through TSE. Finally, the electrical and thermal conductivity of functional prototypes prepared from composite feedstock filaments have been optimized.

## 1. Introduction

Polymeric conductive composites have been explored because of their innumerable advantages over metals in various applications. Power gadgets, electric engines, generators, and thermal exchangers represent some exemplary applications of conductive composites. These composites are lightweight, have better process-capacity, and are shock resistant. Carbon fillers, which have a high thermal and electrical conductivity, can be used to make such polymer composites [1]. Polymers are also used as insulating materials. However, a lot of effort is exerted in order to improve their properties, especially their thermal conductivity. High thermal conductivity is desirable for heat to be efficiently dissipated. Polymers exhibit a low thermal conductivity because of their relatively low atomic density, weak interactions or chemical bonding, and complex crystal structure in their molecular vibrations [2]. The thermal and electrical conductivity of polymers have been traditionally enhanced by the addition of thermally conductive fillers including graphite, carbon black, carbon fibers, Gr, and ceramic or metal particles [3]. Gr is an allotrope of carbon having single layer sp^2^ hybridization arranged in a two-dimensional hexagonal lattice. The material has been of intense interest because of its mechanical, thermal, and electrical properties [4].

Fused deposition modelling (FDM) is one of the additive manufacturing (AM) technologies which works on an additive principle where material in the form of layers is extruded from a nozzle tip [5]. The developments of new materials for FDM are needed to develop in order to increase its application domain in various areas [6,7,8,9,10,11]. The use of recycled plastic waste for the manufacturing of sustainable FDM filaments [12,13,14] is receiving special attention. The use of additive manufacturing techniques for the rapid prototyping of unconventional materials and structures with properties mainly derived from their geometric design, such as cutting-edge reinforcing elements of novel composite materials [15,16,17], and mechanical meta-materials [18,19,20,21,22] is also of increasing interest. A key requirement for any material to be used in FDM is the compatibility of the material with an existing FDM setup without changing the functional hardware/software of the machine. Apart from compatibility, the mechanical, electrical, and thermal properties of the material are also important for its industrial applications [5].

Very few reports of in-house development of an ABS–Gr feedstock filament (from low cost graphite material) for FDM [22,23,24] have appeared. The development of feedstock filament using nanoplatelets of Gr and ABS in various proportions with an aim to improving electrical and thermal properties of filament wire has previously been reported. Commercially available Gr platelets are quite expensive for batch production activities. Polymer composites (PCs) have attracted attention due to the possibility of improving the properties of primary matrices with the addition of small amounts of an appropriate filler. Adding materials such as carbon nanotubes, nanowires, nanoparticles, and Gr to matrices such as polymers, metals, and ceramics via AM has the potential to improve the performances of the resulting components [25,26]. In particular, reinforcing materials have been considered in the form of spherical particles such as titanium dioxide [27] or fumed silica [28], microfibers such as jute fibers [27], short glass fibers [29], and carbon fibers [30,31], nanofibers such as vapor-grown carbon fibers [32], carbon nanotubes [33,34,35], and nanoclays [36]. Recently, Gr has been investigated as a probable reinforcing agent for polymer-based composites. This kind of filler has been used as a multifunctional reinforcement because it possesses a 2D lattice structure resulting in stupendous mechanical, electrical, and thermal properties. The generated composite, ABS reinforced with Gr, shows enhancement of its mechanical properties and electrical and thermal stability [35,36,37,38].

**Figure 1:**
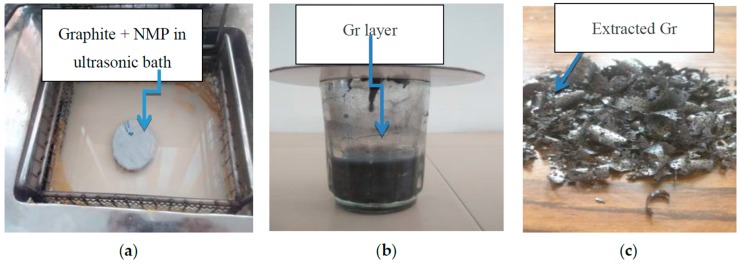
Extraction of Gr. (**a**) Sonication of graphite and NMP; (**b**) Formation of Gr layer; and (**c**) Extracted Gr.

**Figure 2:**
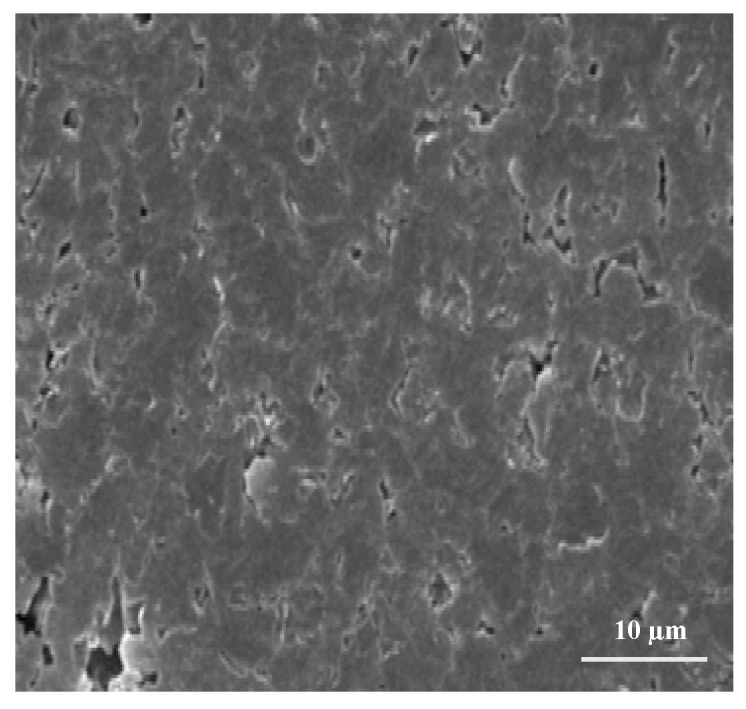
Scanning electron microscopy (SEM) image of extracted Gr.

The present study reveals an effective and efficient method for preparation of a Gr-based polymer composite by direct exfoliation of graphite in organic solvents with addition of naphthalene. The Gr concentration of the dispersion in N-methylpyrrolidone (NMP) may be as high as 0.15 mg/mL. Naphthalene serves as a molecular wedge to intercalate into the edge of graphite, which plays a key role during sonication (see Figure 1a) and significantly improves the production yield of Gr (see Figure 1b,c). Figure 2 shows the scanning electron microscope (SEM) image of the extracted Gr.

As observed from Figure 2, uniform 2D structures have been obtained. This means that the functional group of graphene has not been disturbed. The present study highlights the alternative method for exfoliation of graphite at the lab scale. In this work, out of 50 g graphite, around 3.7 g grapheme was finally extracted. Further chemical analysis may be conducted for ascertaining whether the exfoliation is complete or not. This was not conducted in the present study. The Gr dispersions synthesized by this method can be effectively employed in forming conducting plastic polymer composites by chemical dissolution of Gr (by chemical mixing and by mechanical blending). For chemical mixing, Gr was dispersed in an acetone solution of ABS. The composite slurry was dried in hot air oven at 70 °C for 1.5 h. The resulting lump of composite material was crushed mechanically and fed into TSE. The commercially available Thermo Scientific HAAKE Mini CTW (Make: Karlsruhe, Germany) was used for feedstock filament preparation. The screw rotation speed of 200 RPMs with a load of 10 kg at 180 °C was regulated in order to obtain a final diameter of the extruded filament of 1.75 ± 0.10 mm. In case of mechanical blending, the Gr and ABS were directly fed into the hopper of the TSE. Finally, the polymer composite filament was prepared and fed to FDM for fabrication of functional prototypes.

## 2. Experimentation

The commercially-available ABS was procured from local market (Batra Polymers, Ludhiana, India) having melt flow index (MFI) of 2.9 g/10 min as per the ASTM D 1238-73 standard [39]. The graphite powder (thermal conductivity: 2–90 W/m K) was supplied by Bharat Graphite Pvt. Ltd. (Ludhiana, India). NMP and acetone acted as dissolute and was supplied by Saiteja Chemicals, Hyderabad, India. The acetone acts as a polar aprotic organic solvent that can generally solvate a wide variety of polymers. This solvent can make a dilute ABS solution that can use the intermolecular forces to bond the plastic polymer to Gr. In order to prepare conductive polymer, the Gr was added to the ABS matrix. For addition of Gr in ABS, the MFI values were calculated (see Table 1).

Based upon Table 1, the preliminary experiment was conducted with 25 g Gr blended with 75 g ABS and 40 g acetone (for chemical + mechanical mixing). The resulting weight proportion of Gr has been calculated by using following formula:

(1) $$\mathrm{Gr}=\left[\frac{25\;g\;\left(\mathrm{Gr}\right)}{75\;g\;\left(\mathrm{ABS}\right)+40\;g\;\left(\mathrm{Acetone}\right)}\times 100\right]\;w/w\%=21.7\%$$

Similarly, for another proportion of 90 g ABS and 10 g Gr dispersed in 40 g acetone, the resulting weight proportion of Gr is 7.69%. Finally, these two proportions were taken to conduct the further experimentation.

### 2.1. Thermal Conductivity Measurement

**Figure 3:**
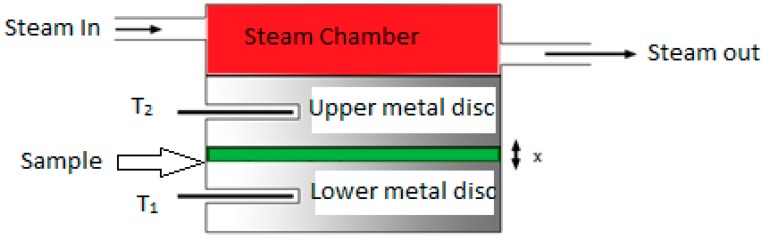
Lee’s disc apparatus for measurement of thermal conductivity.

Figure 3 shows the lab apparatus used to measure thermal conductivity by Lee’s disc method [40]. It consists of two metallic discs and a deep hollow cylinder (steam chamber). The disc has inlet and outlet tubes for steam. In addition, it has radial holes to insert thermometers. The sample is placed within the discs; the upper disc is connected to the hot chamber with the steam inlet. When steam is passed through the cylindrical vessel, a steady state is reached. At the steady state, heat conducted through the bad conductor is equal to heat radiated from the Lees disc. The chamber was set at 70 °C for 2 h.

The thermal conductivity (*k*) of the sample was calculated using Equation (2) [24].

(2) $$k=\frac{\mathrm{mc}\left(\frac{\mathrm{dT}}{\mathrm{dt}}\right)x}{A\left(t_{2}-t_{1}\right)}$$

where:

*k*—Thermal conductivity of the sample
  A—Cross sectional area
  t_2_ − t_1_—Temperature difference across samples
  x—Thickness of the sample
  m—Mass of the disc
  c—Specific heat capacity of disc

The rate of cooling/temperature gradient (dT/dt) has been calculated by plotting the graph of temperature and time.

### 2.2. Electrical Conductivity Test

In order to calculate electrical conductivity, Ohm’s law was used. According to the law, at a steady temperature, the current flowing through a settled straight resistance specifically corresponds to the voltage, and, furthermore, is inversely related to the resistance. This relation between the voltage, current, and resistance forms the basis of Ohm’s Law and is demonstrated as follows.

(3) $$\mathrm{Current}\left(I\right)=\frac{\mathrm{Voltage}\left(V\right)}{\mathrm{Resistance}\left(R\right)},\;\mathrm{in}\;\mathrm{Amperes}\;\left(A\right)$$

**Figure 4:**
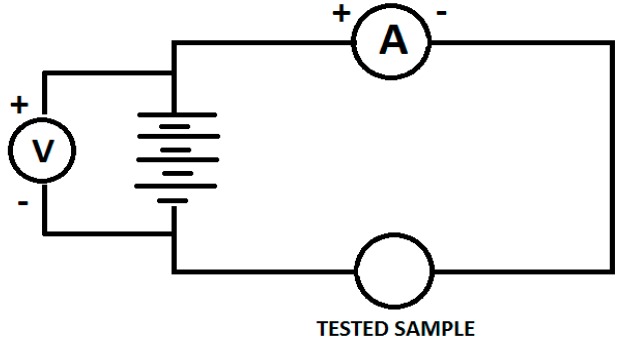
Schematic for Calculation of Electrical conductivity.

In order to evaluate electrical conductivity, 12 V supply is connected across the ammeter and the sample. When the voltage passes across the sample, the ammeter provides a value in amperes (see Figure 4). The values of voltmeter and ammeter were then used to measure the resistance across the sample using Ohm’s law.

Further, the cross-sectional area and length of the sample was measured using digital Vernier caliper (Make: Mitutoyo, Japan) to measure the resistivity. The Resistivity (ρ) of the sample was calculated as:

(4) $$\rho =\frac{RA}{l}$$

where *R* = Resistance, *l* = length of the sample, *A* = cross sectional area of sample.

It is assumed that the current is uniform over the cross-range of the wire. The inverse of resistivity is called conductivity.

Electrical conductivity, σ = $\frac{1}{\rho }$ in S/m (Siemens/meter).

### 2.3. Printing of Functional Prototypes

**Figure 5:**
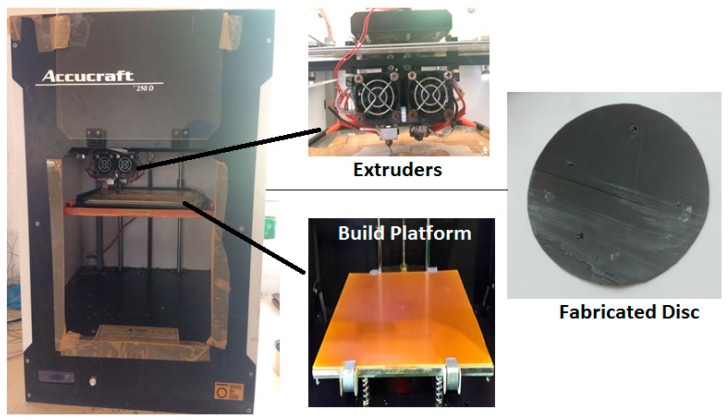
Preparation of functional prototype on FDM.

3D printed specimens were prepared on Accucraft i250 FDM printer (Make: DivideByZero, Mumbai, India) with the extruded filaments. Figure 5 shows a disc prepared as a functional prototype.

For printing of functional prototypes, commercially available Slic3r software (Slic3r is licensed under the GNU Affero General Public License, version 3, Italy) was used. The fixed parameters were honeycomb pattern, nozzle diameter 0.4 mm, layer height 0.4 mm, nozzle temperature 230 °C, and bed temperature 55 °C. Table 2 shows the variable input parameters.

Based upon Table 2, Taguchi L8, Orthogonal array has been selected. Table 3 shows control log of experiment.

Based upon Table 3, electrical and thermal conductivity has been measured. The experiment was repeated three times in order to reduce the experimentation error.

## 3. Results and Discussion

Based upon Table 3, Table 4 shows signal to noise ratio (SN) analysis for electrical conductivity.

**Figure 6:**
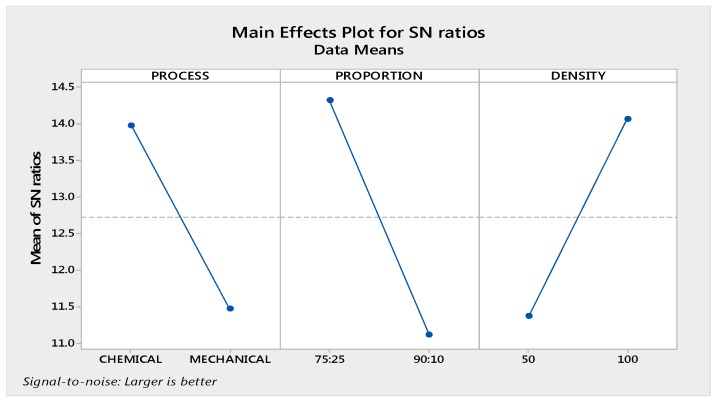
Main effects for signal-to-noise SN ratios of electrical conductivity.

The obtained results for electrical conductivity were calculated for the ‘larger is better’ case by using Minitab17 software (Minitab Ltd., Coventry, UK). Figure 6 shows the main effect plot for the SN ratio for electrical conductivity. As observed from Figure 4, the best setting of input parameters for electrical conductivity is a chemical dissolute sample with proportion 75:25 and in-fill density of 100%.

The results are obvious because chemically blended composites with a high proportion of Gr and high in-fill density must have high electrical conductivity. Further, Table 5 and Table 6 respectively shows the percentage contribution of input parameters based upon analysis of variance (ANOVA) and their rankings.

Residual error was obtained as 4.10% with maximum contribution of proportion, i.e., 41.44% predicts the model for electrical conductivity of a chemically dissolute sample with proportion 75:25 and in-fill density of 100% as having high accuracy. The results are at 95% confidence level.

### 3.1. Optimization of Electrical Conductivity

The optimum value has been predicted by using following formula:

η_opt_ = m + (m_A1_ − m) + (m_B1_ − m) + (m_C2_ − m) (5)

where,

m = overall mean of SN data
  m_A1_= mean of SN data for process at level 1
  m_B1_ = mean of SN data for proportion at level 1
  m_C2_ = mean of SN data for density at level 2
  y_opt_^2^ = (1/10)^ηopt/10^ for properties, lesser is better
  y_opt_^2^ = (10)^ηopt/10^ for properties, Larger is better

Overall mean of SN ratio (m) was taken from Minitab 17.

m = 12.7(6)

Now, from response table of signal to noise ratio, m_A1_ = 13.97, m_B1_ = 14.32, m_C2_ = 14.07.

From here,

η_opt_ = m + (m_A1_ − m) + (m_B1_ − m) + (m_C2_ − m) (7)

η_opt_ = 16.96 db (8)

Now,

y_opt_^2^ = (10)^ηopt/10^ (9)

y_opt_^2^ = (10)^16.96/10^ (10)

y_opt_ = 7.05 S-m (11)

So, the optimum calculated electrical conductivity is 7.05 S-m. The observed value at these setting is 7.29 S-m.

**Figure 7:**
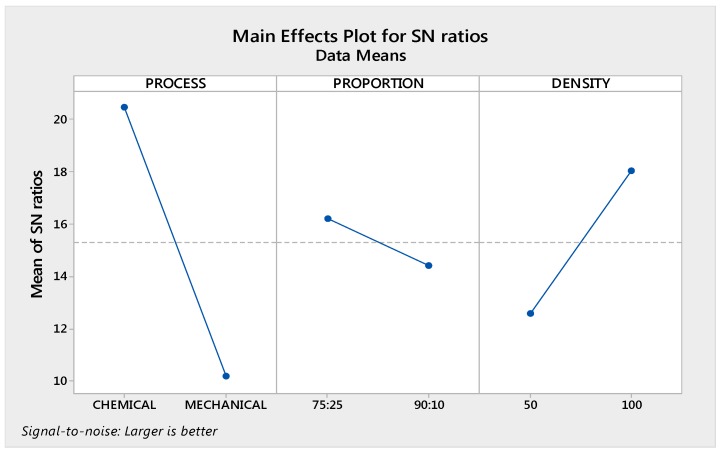
Main effects graph for SN ratios of thermal conductivity.

Similarly, based upon Table 3 and Table 7, Figure 7 shows analysis of thermal conductivity.

Table 8 shows the percentage contribution of input parameters based upon analysis of variance (ANOVA). Further based upon Table 8, rankings of input parameters based upon their contribution for thermal conductivity is shown in Table 9.

### 3.2. Optimization of Thermal Conductivity

The optimum value has been predicted by using following formula:

η_opt_ = m + (m_A1_ − m) + (m_B1_ − m) + (m_C2_ − m) (12)

where,

m = overall mean of SN data
  m_A1_ = mean of SN data for process at level 1
  m_B1_ = mean of SN data for proportion at level 1
  m_C2_ = mean of SN data for density at level 2
  y_opt_^2^ = (1/10)^ηopt/10^ for properties, lesser is better
  y_opt_^2^ = (10)^ηopt/10^ for properties, Larger is better

Adding up, Overall mean of SN ratio (m) was taken from Minitab17.

m = 15.3(13)

Now, from response table of signal to noise ratio, m_A1_ = 20.45, m_B1_ = 16.20, m_C2_ = 18.04. From here,

η_opt_ = m + (m_A1_ − m) + (m_B1_ − m) + (m_C2_ − m) η_opt_ = 24.09 (14)

Now,

y_opt_^2^ = (10)^ηopt/10^ y_opt_ = 16.01 W/mK (15)

The calculated value for thermal conductivity is 16.01 W/mK and observed value is 17.60 W/mK.

### 3.3. Optical Micrograph Observations for Porosity

**Figure 8:**
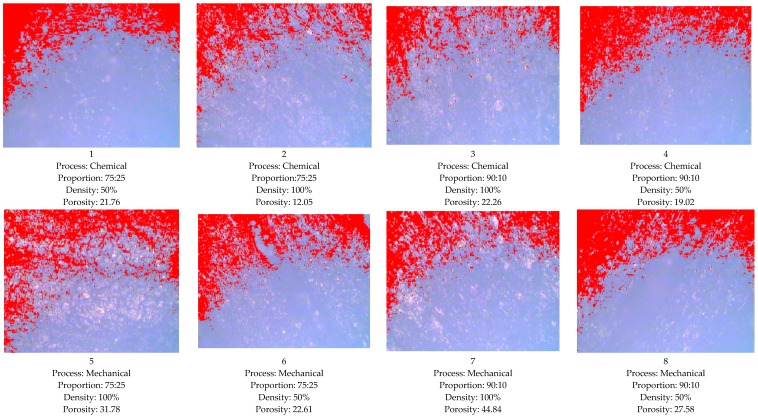
Optical micrographs for ABS–Gr chemically and mechanically blended samples (100×).

Further based upon Table 3, the samples prepared were examined for porosity by optical microscopy at 100× (see Figure 8). For measurement of porosity, the ASTM E2015-04 and ASTM B276 standards were adopted. The commercial software “MIAS” has been used to provide digital output in the form of porosity by converting the image captured into grey-scale. It should be noted that surface roughness can be calculated by this optical way, but this has not been measured in this study. As observed from Figure 8 for sample 2, minimum porosity was observed, which may have contributed to better thermal and electrical properties. The shore D hardness for sample number 2 was measured, and comes out to be 66.3, and for the highest porosity sample, number 7, it comes out to be 59.6. This is counter-verification of the fact that with the better thermal and electrical properties of sample number 2, mechanical properties were also improved by addition of Gr in ABS matrix.

## 4. Conclusions

The FDM feedstock filament with an ABS–Gr matrix has been successfully prepared by exfoliation of graphite at the lab scale. The blending of Gr in ABS has been processed by two methods, mechanical mixing and chemical + mechanical mixing. Finally, the feedstock filament has been successfully used for preparing functional prototypes. The results of the present case study suggest that the electrical and thermal conductivity and mechanical properties of the functional prototypes have been improved. The proportion of Gr in the ABS matrix is the significant parameter which influences the electrical conductivity, followed by the in-fill density and the process used for blending. Whereas for thermal conductivity, the process used for blending (chemical and mechanical mixing), followed by in-fill density and proportion of Gr in the ABS matrix are significant parameters. The Gr-blended ABS specimens with improved mechanical, thermal, and electrical properties can be used for a number of engineering applications. Their association with recycled materials for the manufacturing of innovative, sustainable composites awaits attention [41,42].

## Figures and Tables

**Table 1:** Melt flow index (MFI) of acrylonitrile-butadiene-styrene (ABS) blended with Gr as per ASTM D 1238-73.

ABS:Gr (ByWeight)	Mechanically Blended MFI in g/10 min	Chemically Blended MFI in g/10 min
50:50	0.82	1.63
60:40	1.62	2.27
70:30	2.20	3.20
80:20	2.46	3.94
90:10	2.51	4.12

**Table 2:** Input parameters.

Serial No.	Input Parameters	Levels
1	Infilldensity	50%, 100%
2	Blending process	Mechanical, Chemical + mechanical
3	Proportion of ABS:Gr (weight %)	75:25, 90:10

**Table 3:** Control log of experiment.

Blending Process	Proportion (by Weight)	Infill Density (Percentage)
Chemical	75:25	50
Chemical	75:25	100
Chemical	90:10	50
Chemical	90:10	100
Mechanical	75:25	50
Mechanical	75:25	100
Mechanical	90:10	50
Mechanical	90:10	100

**Table 4:** Electrical conductivity of tested sample with SN Ratios.

Blending Process	Proportion (by Weight)	Infill Density (Percentage)	Electrical Conductivity (S-m)	SN Ratio
Chemical	75:25	50	4.82	13.6609
Chemical	75:25	100	7.29	17.2546
Chemical	90:10	50	3.50	10.8814
Chemical	90:10	100	5.07	14.1002
Mechanical	75:25	50	4.30	12.6694
Mechanical	75:25	100	4.85	13.7148
Mechanical	90:10	50	2.60	8.2995
Mechanical	90:10	100	3.63	11.1981

**Table 5:** ANOVA for SN ratios of electrical conductivity.

Source	DF	Seq SS	Adj SS	Adj MS	F	P	Percentage Contribution
Process	1	12.538	12.538	12.538	24.64	0.008	25.29
Proportion	1	20.546	20.546	20.5459	40.37	0.003	41.44
Density	1	14.463	14.463	14.4629	28.42	0.006	29.17
Residual Error	4	2.036	2.036	0.5089			4.10
Total	7	49.582					

DF: Degree of freedom, Seq SS: Sequential sums of squares, Adj SS: Adjusted sum of squares, Adj MS: Adjusted mean squares.

**Table 6:** Ranking of input process parameters for electrical conductivity.

Level	Process	Proportion	Density
1	13.97	14.32	11.38
2	11.47	11.12	14.07
Delta	2.50	3.21	2.69
Rank	3	1	2

**Table 7:** Thermal conductivity of tested sample with SN ratios.

Blending Process	Proportion (by Weight)	Infill Density (Percentage)	Thermal Conductivity (W/mK)	SN Ratio
Chemical	75:25	50	8.85	18.9389
Chemical	75:25	100	17.60	24.9103
Chemical	90:10	50	6.36	16.0691
Chemical	90:10	100	12.43	21.8894
Mechanical	75:25	50	2.40	7.6042
Mechanical	75:25	100	4.65	13.3491
Mechanical	90:10	50	2.41	7.6403
Mechanical	90:10	100	3.99	12.0195

**Table 8:** ANOVA for SN ratios of thermal conductivity.

Source	DF	Seq SS	Adj SS	Adj MS	F	P	Percentage Contribution
Process	1	212.124	212.124	212.124	245.14	0.000	75.20
Proportion	1	6.451	6.451	6.451	7.46	0.052	2.28
Density	1	60.037	60.037	60.037	69.38	0.001	21.28
Residual Error	4	3.461	3.461	0.865			1.22
Total	7	282.074					

DF: Degree of freedom, Seq SS: Sequential sums of squares, Adj SS: Adjusted sum of squares, Adj MS: Adjusted mean squares.

**Table 9:** Ranking of input process parameters for thermal conductivity.

Level	Process	Proportion	Density
1	20.45	16.20	12.56
2	10.15	14.40	18.04
Delta	10.30	1.80	5.48
Rank	1	3	2

## Supplementary Materials

Supplementary File 1
