# Supplementary material for: Investigations for Thermal and Electrical Conductivity of ABS-Graphene Blended Prototypes
Source: Materials (Basel). 2017 Jul 31;10(8):881. doi: 10.3390/ma10080881 (PMC5578247; doi:10.3390/ma10080881)
Supplement: Supplementary File 1 [file materials-10-00881-s001.pdf]

Point by Point Reply to Reviewers for "Investigations for thermal and electrical conductivity of ABS-Graphene blended prototypes" by Rupinder Singh, Gurleen S. Sandhu, Rosa Penna and Ilenia Farina.

We thank the reviewers for their time and their attentive review. Please find below our replies.

Reviewer 1

Queries raised	Response
Fig.1 is confusing; can you provide electron microscopy images showing structure of the materials?	The SEM image has been incorporated. Please see Fig.2
Fig.2 is not clear, either move to supplementary or with better illustration showing how thermal conductivity is measured.	See Fig.3 for detailed description.
Most of experiments details are missing.	Modifications have been incorporated and put in red colour.
Fig.6, how optical microscopy can measure porosity? Based on my understanding, contract of these images can also present roughness of a surface. Please provide more details.	For measurement of porosity ASTM E2015 – 04 and ASTM B276 standard has been adopted. The commercial software "MIAS" provides digital output in form of porosity by converting the image captured in to gray scale. Also surface roughness can be calculated by this optical way, but the same has not been measured in this study.

Reviewer 2

Queries raised	Response
This manuscript reports the electrical and thermal properties of graphite/graphene containing ABS.<br>The nature of the compositions Obtained/tested is not clear.<br><br>Was the exfoliation of graphite complete? How was this demonstrated? If not, what was the extent of exfoliation, i.e., how much graphene was present in the compositions used?<br><br>Are the compositions genuine composites or simply filled polymers? No evidence for enhanced physical properties for the compositions is presented.<br><br>The discussion of the preparation<br>and properties (exact nature) of the compositions generated needs to be expanded. The thermal/electrical properties obviously will vary depending on the nature of the material being tested.	Fig. 2 shows the SEM image of extracted graphene. As observed from Fig. 2 uniform two dimensional structures has been obtained. This means that the functional group of graphene has not been disturbed.<br><br>The present study highlights the alternative method for exfoliation of graphite at lab scale. For this work, out of 50g graphite around 3.7g graphene was finally extracted. Further chemical analysis may be conducted for ascertaining whether the exfoliation is complete or not. This was not conducted in the present study.<br><br>The composite so prepared has been processed through two methods.<br>(a) Chemical +Mechanical Blending through twin screw extrusion<br>(b) Mechanical Blending through twin screw extrusion<br>In the present study both these methods have been used for preparation of composite feed stock filament and also their comparative study has been performed. It is further submitted that the calculated porosity is the direct reflection of enhanced mechanical properties. Further the shore hardness of the specimens have been measured and put into the manuscript.<br><br>Discussion has been extended and put in red colour.
The manuscript will need substantial rewriting for clarity and readability. Corrections are penciled-in directly on pages of the manuscript attached. These are indicative of the kinds of changes needed throughout.	Modifications have been incorporated as recommended and put in red colour.
